# Supplementary material for: Association between Endometriosis and Delivery Outcomes: A Systematic Review and Meta-Analysis
Source: Biomedicines. 2022 Feb 17;10(2):478. doi: 10.3390/biomedicines10020478 (PMC8962356; doi:10.3390/biomedicines10020478)
Supplement: Supplementary file 1 [file biomedicines-10-00478-s001.zip › BM_CD_SR_Metadata table_123121.pdf]

Supplemental File S1. The search strategy.

PubMed

#1 Endometriosis [MeSH]

#2 endometriosis [TIAB] OR endometrioma [TIAB] OR chocolate cyst [TIAB]

#3 adhesion [TIAB] OR “ectopic endometri*” [TIAB]

#4 peritoneal [TIAB] OR abdominal [TIAB] OR pelvic [TIAB] OR Douglas [TIAB] OR “uterine wall” [TIAB] OR “anterior wall” [TIAB] OR “posterior wall” [TIAB] OR abdominal [TIAB] OR ovary [TIAB] OR extrauterine [TIAB] OR extraovarian [TIAB] OR cul-de-sac [TIAB] OR adnexal [TIAB]

#5 #3 AND #4

#6 #1 OR #2 OR #5

#7 Pregnancy [MeSH] OR Birth intervals[MeSH] OR pregnanc*[TIAB] OR pregnant[TIAB] OR gravid*[TIAB] OR obstet*[TIAB] OR postpartum*[TIAB] OR birth[TIAB] OR fetus*[TIAB] OR foetus*[TIAB] OR fetal[TIAB] OR foetal[TIAB] OR gestation[TIAB] OR gestations[TIAB]

#8 #6 AND #7

#9 Placenta previa [MeSH] OR “placenta previa” [TIAB] OR "low lying placenta" [TIAB] OR Fetal growth retardation [MeSH] OR “fetal growth restriction” [TIAB] OR “intrauterine growth restriction” [TIAB] OR “intrauterine growth retardation” [TIAB] OR Pre-Eclampsia [MeSH] OR preeclampsia [TIAB] OR “pre-eclampsia” [TIAB] OR Postpartum Hemorrhage [MeSH] OR “postpartum hemorrhage” [TIAB] OR Obstetric Labor Complications [MeSH] OR “obstetric labor complications” [TIAB] OR “labor complications” [TIAB] OR Abruptio Placentae [MeSH] OR “abruptio placentae” [TIAB] OR “placental abruption” [TIAB] OR “placental abruptions” [TIAB] OR Uterine Rupture [MeSH] OR “uterine rupture” [TIAB] OR “uterine ruptures” [TIAB] OR Dystocia [MeSH] OR “dystocia” [TIAB] OR “dystocias” [TIAB] OR Breech Presentation [MeSH] OR “breech presentation” [TIAB] OR “breech fetal presentation” [TIAB] OR Placenta accreta [MeSH] OR “placenta accreta” [TIAB] OR “placenta accreta spectrum” [TIAB] OR “placenta increta” [TIAB] OR “placenta percreta" [TIAB]

#10 Cesarean section [MeSH] OR cesarean section [TIAB] OR Cesarean delivery [TIAB] OR abdominal delivery [TIAB] OR “C section” [TIAB]

#11 #9 OR #10

#12 #8 AND #11

Scopus

#1 TITLE-ABS-KEY (endometriosis)

#2 TITLE-ABS-KEY (endometrioma OR “chocolate cyst”)

#3 TITLE-ABS-KEY (adhesion OR “ectopic endometri*”)

#4 TITLE-ABS-KEY (peritoneal OR abdominal OR pelvic OR douglas OR “uterine wall” OR “anterior wall” OR “posterior wall” OR ovary OR extrauterine OR extraovarian OR cul-de-sac OR adnexal)

#5 #3 AND #4

#6 #1 OR #2 OR #5

#7 TITLE-ABS-KEY (pregnancy OR pregnanc* OR pregnant OR gravid* OR obstet* OR postpartum* OR birth OR fetus* OR foetus* OR fetal OR foetal OR gestation OR gestations)

#8 #6 AND #7

#9 TITLE-ABS-KEY (“placenta previa” OR "low lying placenta" OR “fetal growth restriction” OR “intrauterine growth restriction” OR “intrauterine growth retardation” OR preeclampsia OR “pre-eclampsia” OR “postpartum hemorrhage” OR “obstetric labor complications” OR “labor complications” OR “abruptio placentae” OR “placental abruption” OR “placental abruptions” OR “uterine rupture” OR “uterine ruptures” OR “dystocia” OR “dystocias” OR “breech presentation” OR

“breech fetal presentation” OR “placenta accreta” OR “placenta accreta spectrum” OR “placenta increta” OR “placenta percreta")
#10 TITLE-ABS-KEY (“cesarean section” OR “cesarean delivery” OR “abdominal delivery” OR “C section”)
#11 #9 OR #10
#12 #8 AND #11

Cochrane
#1 Endometriosis [MeSH]
#2 endometriosis OR endometrioma OR chocolate cyst
#3 adhesion OR “ectopic endometri*”
#4 peritoneal OR abdominal OR pelvic OR Douglas OR “uterine wall” OR “anterior wall” OR “posterior wall” OR abdominal OR ovary OR extrauterine OR extraovarian OR cul-de-sac OR adnexal
#5 #3 AND #4
#6 #1 OR #2 OR #5
#7 Pregnancy [MeSH]
#8 Birth intervals [MeSH]
#9 pregnanc* OR pregnant OR gravid* OR obstet* OR postpartum* OR birth OR fetus* OR foetus* OR fetal OR foetal OR gestation OR gestations
#10 #7 OR #8 OR #9
#11 #6 AND #10
#12 Placenta previa [MeSH]
#13 Fetal growth retardation [MeSH]
#14 Pre-Eclampsia [MeSH]
#15 Abruptio Placentae [MeSH]
#16 Uterine Rupture [MeSH]
#17 Breech Presentation [MeSH]
#18 Placenta accreta [MeSH]
#19 Dystocia [MeSH]
#20 Obstetric Labor Complications [MeSH]
#21 Postpartum Hemorrhage [MeSH]
#22 “placenta previa” OR "low lying placenta" OR “fetal growth restriction” OR “intrauterine growth restriction” OR “intrauterine growth retardation”
#23 preeclampsia OR “pre-eclampsia” OR “postpartum hemorrhage” OR “obstetric labor complications” OR “labor complications” OR “abruptio placentae” OR “placental abruption” OR “placental abruptions”
#24 “uterine rupture” OR “uterine ruptures” OR “dystocia” OR “dystocias” OR “breech presentation” OR “breech fetal presentation” OR “placenta accreta” OR “placenta accreta spectrum”
#25 Cesarean section [MeSH]
#26 cesarean section OR Cesarean delivery OR abdominal delivery OR “C section”
#27 #12 OR #13 OR #14 OR #15 OR 16 OR #17 OR #18 OR #19 OR #20 OR #21 OR #22 OR #23 OR #24 OR #25 OR #26
#28 #11 AND #27

Supplemental File S2. Metadata of included studies.

Author	Year	Area	Study period	Study type	Match	Total No.	Endo No.	Control No.	Instrumental delivery	CD	PPH	Definition of Endo
Berlanda N [24]	2021	ITA	2017-2018	Retro	Yes	894	297	597	--	Yes	--	Clinical or surgical diagnosis
Lin S [25]	2020	CHN	2013-2016	Retro	--	246	82	164	--	Yes	Yes	Past surgical history
Wu J [26]	2020	CHN	2006-2017	Retro	--	7086	1111	5975	--	Yes	--	Past surgical history
Yi KW [27]	2020	KOR	2007-2015	Population	Yes	1938424	44428	1893996	--	Yes	Yes	ICD-10 code
Porpora MG [28]	2020	ITA	2013-2019	Retro	--	425	145	280	--	Yes	Yes	Clinical or surgical diagnosis
Miura M [29]	2019	JPN	2010-2017	Retro	--	2769	80	2689	Yes	Yes	Yes	Clinical or surgical diagnosis
Uccella S [30]	2019	ITA	2011-2014	Retro	--	1808	118	1690	Yes	Yes	Yes	Past surgical history
Shmueli A [31]	2019	ISR	2007-2014	Retro	Yes	61535	135	61400	Yes	Yes	Yes	ICD-10 code
Nirgianakis K [32]	2018	CHE	2004-2015	Retro	Yes	248	62	186	Yes	Yes	Yes	Past surgical history
Chen I [33]	2018	CAN	2003-2013	Retro	Yes	52202	469	51733	--	Yes	Yes	ICD-10 code
Tzur T [34]	2018	ISR	1988-2013	Retro	Yes	502	35	467	--	Yes	Yes	Past surgical history
Li H [35]	2017	CHN	2011-2013	Retro	Yes	375	75	300	--	Yes	Yes	Past surgical history
Berlac JF [36]	2017	DNK	1977-2014	Population	Yes	1091251	19331	1071920	Yes	Yes	Yes	ICD-10 code
						1075846	3926*	1071920	Yes	Yes	Yes	Past surgical history
Saraswat L [37]	2017	UK	1981-2010	Population	Yes	10939	4232	6707	Yes	Yes	Yes	Past surgical history
Mannini L [38]	2017	ITA	2009-2014	Retro	--	786	262	524	--	Yes	Yes	Past surgical history
Harada T [39]	2016	JPN	2011	Retro	--	9186	330	8856	--	Yes	--	Clinical or surgical diagnosis
Glavind MT [40]	2017	DNK	1989-2013	Population	Yes	82793	1719	81074	--	Yes	Yes	ICD-10 code
Benaglia L [41]	2016	ITA	2008-2014	Retro	--	478	239	239	--	Yes	--	Clinical or surgical diagnosis
Exacoustos C [84]	2016	ITA	2011-2015	Retro	--	341	41	300	Yes	Yes	--	Past surgical history
Jacques M [43]	2016	FRA	2009-2104	Retro	Yes	223	113	113	--	Yes	Yes	Clinical or surgical diagnosis
Baggio S [44]	2015	ITA	1996-2007	Retro	--	123	30	93	--	Yes	--	Past surgical history
Lin H [45]	2015	CHN	1995-2009	Retro	Yes	498	249	249	--	Yes	--	Past surgical history
Conti N [46]	2015	ITA	--	Retro	--	2239	316	1923	Yes	Yes	Yes	Past surgical history
Mekaru K [47]	2014	JPN	1995-2011	Retro	--	88	40	48	--	Yes	--	Past surgical history
Benaglia L [48]	2012	ITA	2005-2009	Retro	Yes	191	61	130	--	Yes	--	Clinical diagnosis
Healy DL [49]	2010	AUS	1991-2004	Retro	Yes	6730	1265	5465	--	--	Yes	--
Stephansson O [50]	2009	SWE	1992-2006	Population	Yes	1442675	13090	1429585	--	Yes	--	ICD-10 code
Kortelahti M [51]	2003	FIN	1994-2000	Retro	Yes	274	137	137	Yes	Yes	--	Past surgical history

* Restricted to women with histologically diagnosed endometriosis. Some values listed above might be slightly different from the original values, as estimated by the authors. Abbreviations: Abbreviations: No., number of cases; Endo, endometriosis; CD, cesarean delivery; PPH, postpartum hemorrhage; Retro, retrospective study; Prosp, prospective study; Population, population-based study; Match, patient background matched study; --, not applicable; ICD-10, International Classification of Diseases 10th Revision; ITA, Italy; CHN, China; KOR, Korea; JPN, Japan; CHE, Switzerland; CAN, Canada; ISR, Israel; DNK, Denmark; FRA, France; SWE, Sweden; UK, United Kingdom; FIN, Finland; AUS, Australia.

Supplemental File S3. Metadata of included studies (the rate of instrumental delivery).

Author	Year	Total No.	Endo No.	Control No.	Instrumental delivery				Definition of Endo
					Endo No. (%)	Control No. (%)	OR (95% CI)	adjusted OR (95% CI)	
Miura M [29]	2019	2769	80	2689	7 (8.8)	267 (9.9)	0.87 (0.40-1.91)	--	Clinical or surgical diagnosis
Uccella S [30]	2019	1808	118	1690	7 (5.9)	37 (2.2)	2.82 (1.23-6.46)	--	Past surgical history
Shmueli A [31]	2019	61535	135	61400	7 (5.2)	5206 (8.5)	0.59 (0.28-1.26)	--	ICD-10 code
Nirgianakis K [32]	2018	248	62	186	7 (11.3)	39 (21.0)	0.48 (0.20-1.14)	--	Past surgical history
Berlac JF [36]	2017	1091251	19331	1071920	1507 (7.8)	81049 (7.6)	1.03 (0.98-1.09)	1.2 (1.1-1.3)	ICD-10 code
		1075846	3926*	1071920	355 (9.0)	81049 (7.6)	1.22 (1.09-1.36)	1.6 (1.5-1.8)	Past surgical history
Saraswat L [37]	2017	10939	4232	6707	822 (19.4)	928 (13.8)	1.50 (1.35-1.66)	1.21 (1.08-1.36)	Past surgical history
Exacoustos C [84]	2016	341	41	300	1 (2.4)	19 (6.3)	0.37 (0.05-2.84)	--	Past surgical history
Conti N [46]	2015	2239	316	1923	10 (3.2)	55 (2.9)	1.11 (0.56-2.20)	--	Past surgical history
Kortelahti M [51]	2003	274	137	137	8 (5.8)	11 (8.0)	0.71 (0.28-1.82)	--	Past surgical history

* Restricted to women with histologically diagnosed endometriosis. Some values listed might be slightly different from the original values because the calculation was performed using Revman ver. 5.4.1. Some values listed above might be slightly different from the original values, as estimated by the authors. Abbreviations: No., number of cases; Endo, endometriosis; OR, odds ratio; CI, confidence interval; --, not applicable; ICD-10, International Classification of Diseases 10th Revision.

Supplemental File S4. Metadata of included studies (the rate of cesarean delivery).

Author	Year	Total No.	Endo No.	Control No.	Cesarean delivery Endo No. (%)	Control No. (%)	OR (95% CI)	adjusted OR (95% CI)	Definition of Endo
Berlanda N [24]	2021	894	297	597	122 (41.1)	188 (31.5)	1.52 (1.14-2.02)	1.27 (0.89-1.80)	Clinical or surgical diagnosis
Lin S [25]	2020	246	82	164	49 (59.8)	63 (38.4)	2.38 (1.38-4.09)	--	Past surgical history
Wu J [26]	2020	7086	1111	5975	827 (74.4)	4453 (74.5)	1.00 (0.86-1.15)	--	Past surgical history
Yi KW [27]	2020	1938424	44428	1893996	21488 (48.4)	713042 (37.6)	1.55 (1.52-1.58)	1.33 (1.30-1.35)	ICD-10 code
Porpora MG [28]	2020	425	145	280	51 (35.2)	87 (31.1)	1.20 (0.79-1.84)	--	Clinical or surgical diagnosis
Miura M [29]	2019	2769	80	2689	43 (53.8)	1177 (43.8)	1.49 (0.96-2.33)	--	Clinical or surgical diagnosis
Uccella S [30]	2019	1808	118	1690	49 (41.5)	409 (24.2)	2.22 (1.52-3.26)	--	Past surgical history
Shmueli A [31]	2019	61535	135	61400	87 (64.4)	11402 (18.6)	7.95 (5.58-11.31)	5.01 (3.34-7.52)	ICD-10 code
Nirgianakis K [32]	2018	248	62	186	36 (58.1)	81 (43.5)	1.79 (1.00-3.21)	--	Past surgical history
Chen I [33]	2018	52202	469	51733	175 (37.3)	15527 (30.0)	1.39 (1.15-1.68)	1.08 (0.97-1.20)	ICD-10 code
Tzur T [34]	2018	502	35	467	32 (91.4)	81 (17.3)	50.83 (15.20-170.03)	38.08 (11.04-131.38)	Past surgical history
Li H [35]	2017	375	75	300	32 (42.7)	100 (33.3)	1.49 (0.89-2.50)	1.53 (0.83-2.84)	Past surgical history
Berlac JF [36]	2017	1091251	19331	1071920	4606 (23.8)	90771 (8.5)	3.38 (3.27-3.50)	--	ICD-10 code
		1075846	3926*	1071920	1266 (32.2)	90771 (8.5)	5.14 (4.81-5.50)	--	Past surgical history
Saraswat L [37]	2017	10939	4232	6707	1299 (30.7)	1281 (19.1)	1.88 (1.72-2.05)	1.40 (1.26-1.55)	Past surgical history
Mannini L [38]	2017	786	262	524	149 (56.9)	190 (36.3)	2.32 (1.71-3.14)	--	Past surgical history
Glavind MT [40]	2017	82793	1719	81074	414 (24.1)	11464 (14.1)	1.93 (1.72-2.16)	1.83 (1.60-2.09)	ICD-10 code
Harada T [39]	2016	9186	330	8856	85 (25.8)	1570 (17.7)	1.61 (1.25-2.07)	--	Clinical or surgical diagnosis
Benaglia L [41]	2016	478	239	239	108 (45.2)	106 (44.4)	1.03 (0.72-1.48)	--	Clinical or surgical diagnosis
Exacoustos C [84]	2016	341	41	300	28 (68.3)	130 (43.3)	2.82 (1.40-5.65)	--	Past surgical history
Jacques M [43]	2016	223	110	113	35 (31.8)	17 (15.0)	2.64 (1.37-5.07)	--	Clinical or surgical diagnosis
Baggio S [44]	2015	123	30	93	18 (60.0)	27 (29.0)	3.67 (1.56-8.64)	--	Past surgical history
Lin H [45]	2015	498	249	249	174 (69.9)	121 (48.6)	2.45 (1.70-3.54)	1.93 (1.31-2.84)	Past surgical history
Conti N [46]	2015	2239	316	1923	81 (25.6)	437 (22.7)	1.17 (0.89-1.54)	--	Past surgical history
Mekaru K [47]	2014	88	40	48	13 (32.5)	11 (22.9)	1.62 (0.63-4.16)	--	Past surgical history
Benaglia L [48]	2012	191	61	130	23 (37.7)	44 (33.8)	1.18 (0.63-2.23)	1.25 (0.63-2.50)	Clinical diagnosis
Stephansson O [50]	2009	1442675	13090	1429585	2815 (21.5)	193082 (13.5)	1.75 (1.68-1.83)	1.47 (1.40-1.54)	ICD-10 code
Kortelahti M [51]	2003	274	137	137	43 (31.4)	42 (30.7)	1.03 (0.62-1.73)	--	Past surgical history

* Restricted to women with histologically diagnosed endometriosis. Some values listed might be slightly different from the original values because the calculation was performed using Revman ver. 5.4.1. Some values listed above might be slightly different from the original values, as estimated by the authors. Abbreviations: No., number of cases; Endo, endometriosis; OR, odds ratio; CI, confidence interval; --, not applicable; ICD-10, International Classification of Diseases 10th Revision.

Supplemental File S5. Metadata of included studies (the rate of postpartum hemorrhage).

Author	Year	Total No.	Endo No.	Control No.	PPH				Definition of Endo	Definition of PPH
					Endo No. (%)	Control No. (%)	OR (95% CI)	adjusted OR (95% CI)		
Lin S [25]	2020	246	82	164	7 (8.5)	12 (7.3)	1.18 (0.45-3.13)	--	Past surgical history	--
Yi KW [27]	2020	1938424	44428	1893996	3913 (8.8)	149794 (7.9)	1.12 (1.09-1.16)	1.10 (1.07-1.14)	ICD-10 code	ICD-10 code
Porpora MG [28]	2020	425	145	280	4 (2.8)	3 (1.1)	2.62 (0.58-11.86)	--	Clinical or surgical diagnosis	500 mL VD<br>1000 mL CD
Miura M [29]	2019	2769	80	2689	22 (27.5)	490 (18.2)	1.70 (1.03-2.81)	--	Clinical or surgical diagnosis	800 mL VD<br>1500 mL CD
Uccella S [30]	2019	1808	118	1690	21 (17.8)	413 (24.4)	0.67 (0.41-1.09)	--	Past surgical history	--
Shmueli A [31]	2019	61535	135	61400	6 (4.4)	1166 (1.9)	2.40 (1.06-5.46)	3.69 (1.60-8.53)	ICD-10 code	500 mL VD<br>1000 mL CD
Nirgianakis K [32]	2018	248	62	186	10 (16.1)	16 (8.6)	2.04 (0.87-4.78)	--	Past surgical history	500 mL VD<br>1000 mL CD
Chen I [33]	2018	52202	469	51733	35 (7.5)	3548 (6.9)	1.10 (0.77-1.55)	1.02 (0.74-1.41)	ICD-10 code	ICD-10 code
Tzur T [34]	2018	502	35	467	0 (0)	3 (0.6)	1.87 (0.09-36.90)	--	Past surgical history	--
Li H [35]	2017	375	75	300	18 (24.0)	38 (12.7)	2.18 (1.16-4.09)	2.27 (1.06-4.87)	Past surgical history	--
Berlac JF [36]	2017	1091251	19331	1071920	1698 (8.8)	108161 (10.1)	0.86 (0.82-0.90)	0.95 (0.90-1.00)	ICD-10 code	--
		1075846	3926*	1071920	550 (14.0)	108161 (10.1)	1.45 (1.33-1.59)	0.87 (0.80-0.95)	Past surgical history	--
Glavind MT [40]	2017	82793	1719	81074	159 (9.2)	7637 (9.4)	0.98 (0.83-1.16)	0.95 (0.80-1.14)	ICD-10 code	500 mL
Saraswat L [37]	2017	10939	4232	6707	844 (19.9)	786 (11.7)	1.88 (1.69-2.09)	1.37 (1.16-1.61)	Past surgical history	500 mL VD<br>1000 mL CD
Mannini L [38]	2017	786	262	524	22 (8.4)	49 (9.4)	0.89 (0.52-1.50)	--	Past surgical history	500 mL
Jacques M [43]	2016	223	113	113	10 (8.8)	10 (8.8)	1.00 (0.40-2.50)	--	Clinical or surgical diagnosis	--
Conti N [46]	2015	2239	316	1923	30 (9.5)	164 (8.5)	1.13 (0.75-1.69)	--	Past surgical history	500 mL VD<br>750 mL CD
Healy DL [49]	2010	6730	1265	5465	165 (13.0)	581 (10.6)	1.26 (1.05-1.52)	1.29 (1.06-1.56)	--	500 mL VD<br>750 mL CD

* Restricted to women with histologically diagnosed endometriosis. Some values listed might be slightly different from the original values because the calculation was performed using Revman ver. 5.4.1. Some values listed above might be slightly different from the original values, as estimated by the authors. Abbreviations: No., number of cases; Endo, endometriosis; PPH, postpartum hemorrhage; VD, vaginal delivery; CD, cesarean delivery; OR, odds ratio; CI, confidence interval; --, not applicable; ICD-10, International Classification of Diseases 10th Revision.

Supplemental File S6. Risk of bias assessment for the comparator study.

Authors	Confounding	Selection	Classification of intervention	Deviations from interventions	Missing data	Measurement of outcomes	Reported results	Overall bias
Berlanda N [24]	● (purple)	● (purple)	● (orange)	● (black)	● (purple)	● (purple)	● (orange)	● (purple)
Lin S [25]	● (purple)	● (purple)	● (orange)	● (black)	● (black)	● (red)	● (purple)	● (red)
Wu J [26]	● (purple)	● (purple)	● (orange)	● (black)	● (purple)	● (purple)	● (purple)	● (purple)
Yi KW [27]	● (purple)	● (purple)	● (red)	● (black)	● (purple)	● (red)	● (orange)	● (purple)
Porpora MG [28]	● (purple)	● (red)	● (orange)	● (black)	● (black)	● (purple)	● (purple)	● (purple)
Miura M [29]	● (purple)	● (purple)	● (purple)	● (black)	● (black)	● (purple)	● (purple)	● (purple)
Uccella S [30]	● (purple)	● (purple)	● (purple)	● (black)	● (black)	● (red)	● (purple)	● (purple)
Shmueli A [31]	● (purple)	● (purple)	● (red)	● (black)	● (black)	● (purple)	● (orange)	● (purple)
Nirgianakis K [32]	● (purple)	● (red)	● (orange)	● (black)	● (black)	● (purple)	● (orange)	● (purple)
Chen I [33]	● (purple)	● (purple)	● (red)	● (black)	● (black)	● (purple)	● (purple)	● (purple)
Tzur T [34]	● (red)	● (red)	● (orange)	● (black)	● (black)	● (red)	● (purple)	● (red)
Li H [35]	● (red)	● (red)	● (orange)	● (black)	● (black)	● (red)	● (purple)	● (red)
Berlac JF [36]	● (purple)	● (purple)	● (red)	● (black)	● (black)	● (red)	● (orange)	● (purple)
Saraswat L [37]	● (purple)	● (purple)	● (orange)	● (black)	● (orange)	● (purple)	● (orange)	● (purple)
Mannini L [38]	● (purple)	● (red)	● (orange)	● (black)	● (black)	● (purple)	● (purple)	● (purple)
Harada T [39]	● (purple)	● (purple)	● (purple)	● (black)	● (orange)	● (purple)	● (purple)	● (purple)
Glavind MT [40]	● (purple)	● (purple)	● (purple)	● (black)	● (orange)	● (purple)	● (orange)	● (purple)
Benaglia L [41]	● (purple)	● (red)	● (purple)	● (black)	● (black)	● (purple)	● (purple)	● (purple)
Exacoustos C [84]	● (purple)	● (red)	● (orange)	● (black)	● (black)	● (purple)	● (purple)	● (purple)
Jacques M [43]	● (red)	● (red)	● (purple)	● (black)	● (black)	● (purple)	● (purple)	● (red)
Baggio S [44]	● (red)	● (purple)	● (orange)	● (black)	● (black)	● (red)	● (purple)	● (purple)
Lin H [45]	● (purple)	● (red)	● (orange)	● (black)	● (black)	● (purple)	● (purple)	● (purple)
Conti N [46]	● (red)	● (purple)	● (orange)	● (black)	● (black)	● (purple)	● (purple)	● (purple)
Mekaru K [47]	● (red)	● (red)	● (orange)	● (black)	● (black)	● (purple)	● (purple)	● (red)
Benaglia L [48]	● (purple)	● (red)	● (purple)	● (black)	● (black)	● (purple)	● (purple)	● (purple)
Healy DL [49]	● (purple)	● (purple)	● (red)	● (black)	● (black)	● (purple)	● (purple)	● (purple)
Stephansson O [50]	● (purple)	● (purple)	● (purple)	● (black)	● (purple)	● (purple)	● (orange)	● (purple)
Kortelahti M [51]	● (purple)	● (red)	● (orange)	● (black)	● (black)	● (purple)	● (purple)	● (purple)

Updated and reproduced the data from Am J Obstet Gynecol MFM. 2021;3:100417. Matsuzaki S *et al.* [13].The association of endometriosis with placenta previa and postpartum hemorrhage: a systematic review and meta-analysis / Supplemental Table S3. Risk of bias assessment for the comparator study. Copyright (2021), with permission from Elsevier.

Risk of bias assessment was performed using the Risk Of Bias In Non-randomized Studies–of Interventions tool (ROBINS-I) [20-22].

● (orange) Low risk of bias (the study is comparable to a well-performed randomized trial with regard to this domain)

● (purple) Moderate risk of bias (the study is sound for a non-randomized study with regard to this domain but cannot be considered comparable to a well-performed randomized trial)

- Serious risk of bias (the study has some important problems in this domain)
- Critical risk of bias (the study is too problematic in this domain to provide any useful evidence on the effects of intervention.
- No information on how to base a judgment on the risk of bias for this domain
